# Supplementary material for: Matrix mechanics regulates epithelial defence against cancer by tuning dynamic localization of filamin
Source: Nat Commun. 2022 Jan 11;13:218. doi: 10.1038/s41467-021-27896-z (PMC8752856; doi:10.1038/s41467-021-27896-z)
Supplement: Supplementary file 1 — Supplementary Information [file 41467_2021_27896_MOESM1_ESM.pdf]

Supplementary Information

Matrix mechanics regulates epithelial defence against cancer by tuning dynamic localization of filamin

Pothapragada *et al.*

This document contains:

1. Supplementary Figures 1-6 with respective legends
2. Supplementary Table 1: Composition of compliant polyacrylamide gels with different stiffness.
3. Supplementary Table 2: Antibody information
4. Supplementary Table 3: Plasmid information

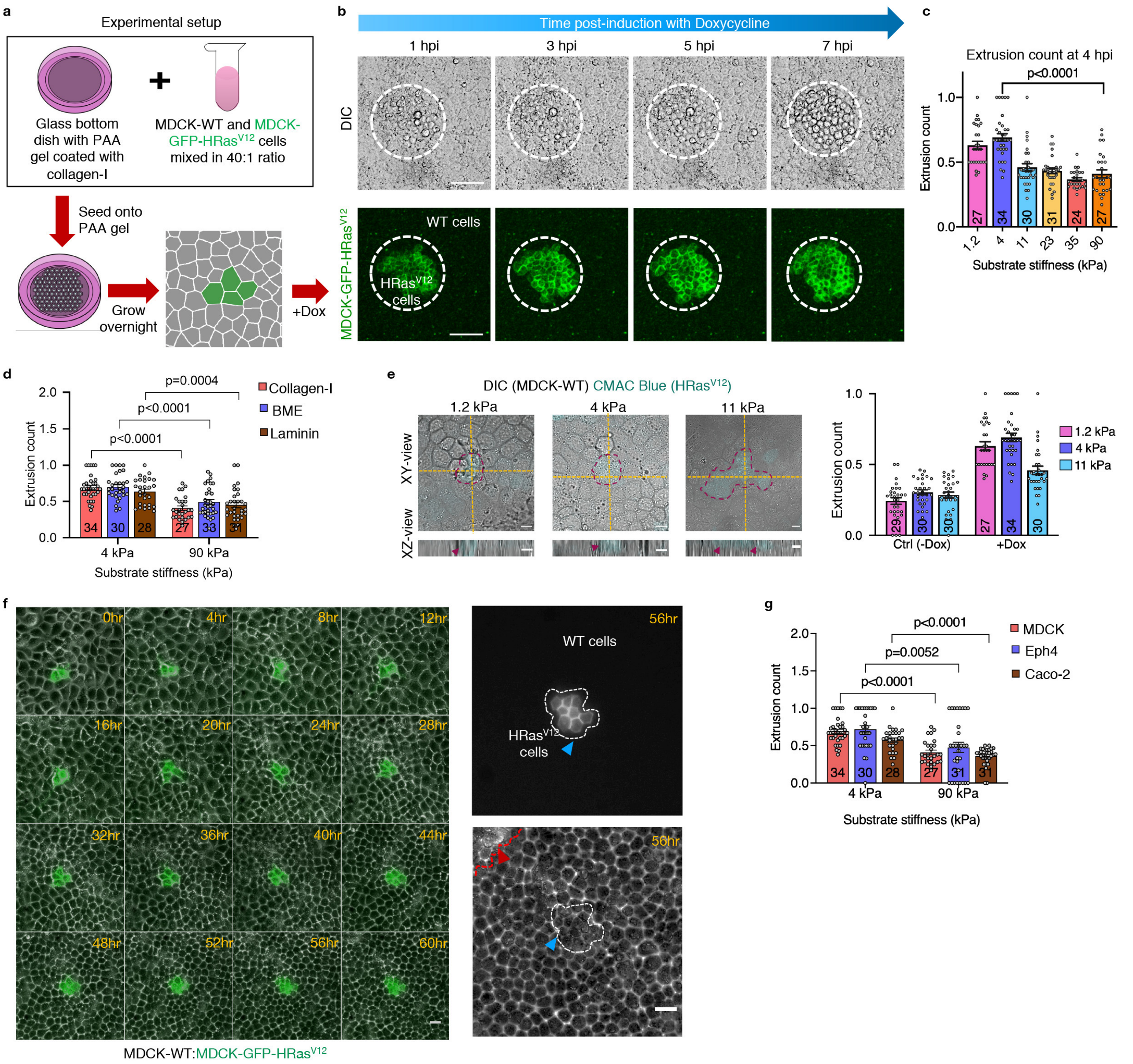

Supplementary Figure 1. Extrusion of MDCK-GFP-HRas^{V12}-transformed cells co-cultured with MDCK-WT cells. **a.** Illustration detailing the used experimental epithelial monolayer model of EDAC. **b.** Representative DIC images of MDCK-WT:MDCK-GFP-HRas^{V12} mosaic monolayer (*top row*), fluorescence images of GFP-HRas^{V12} expressing cells (*bottom row*). From left to right: snapshots of GFP-HRas^{V12} expressing cells at different time points post-induction with doxycycline. hpi: hour post-induction. One representative extruding colony of transformed cells encircled (dotted-white circle). Scale bars = 60μm. **c.** Scatter bar plots depicting the fraction of GFP-HRas^{V12} expressing colonies extruded over substrates of varying stiffness at 4 hpi. The number of colonies counted is indicated inside each bar. Data are mean±s.e.m. collected over 3 independent biological replicates. Statistical significance was assessed using Mann-Whitney t-test (two-tailed). p=3.5771e-07 **d.** Scatter bar plots depicting the fraction of GFP-HRas^{V12}-expressing colonies extruded over soft (4 kPa) and stiff (90 kPa) ECM at 4 hpi for MDCK mosaic cultures grown on basement membrane extract-coated and Laminin-coated gels. **e.** Representative DIC images of MDCK-WT:MDCK-HRas^{V12} mosaic monolayer cultured on soft substrates (*left-right*, 1.2, 4 and 11 kPa) overlapped with CellTracker CMAC-Blue labelled, uninduced MDCK-HRas^{V12} cells colour-coded in cyan and outlined in magenta. Non-extruded HRas^{V12} cells remain in plane with other cells, as evident in the XZ-plane. (*Bottom panels, magenta arrowheads*) The yellow-dotted lines visually guide epithelium surface in XZ-plane. (*Right*) Scatter bar plots depicting the fraction of uninduced-MDCK-HRas^{V12} colonies extruded over soft substrates compared with extrusion fraction of induced GFP-HRas^{V12} expressing cells. **f.** Representative live imaging time stamps for a competing mosaic monolayer of MDCK-WT and MDCK-GFP-HRas^{V12} seeded on 90 kPa PAA gel and observed upto 60 hours. Extrusion is hindered on stiff ECM and transformed cells (*white outline, blue arrowhead*) are seen to undergo continuous division while normal cells undergo crowding-induced extrusion (*red outline and arrowhead*). Scale bar = 20 μm. **g.** Scatter bar plots depicting the fraction of GFP-HRas^{V12}-expressing colonies extruding from GFP-

$HRas^{V12}$-transfected Eph4 or Caco2 monolayers. **(d,e,g)** Numbers inside the bar plots indicate sample size. Data are mean±s.e.m. collected over 3 independent biological replicates. Statistical significance was assessed using Mann-Whitney t-test (two-tailed, **d**) or unpaired student t-test with Welch's correction (two-tailed, **g**). Significance values: p=2.8490e-08 (Collagen-I, MDCK), p = 2.90888e-07 (Caco2). Source data (**c,d,e,g**) are provided as a Source Data file.

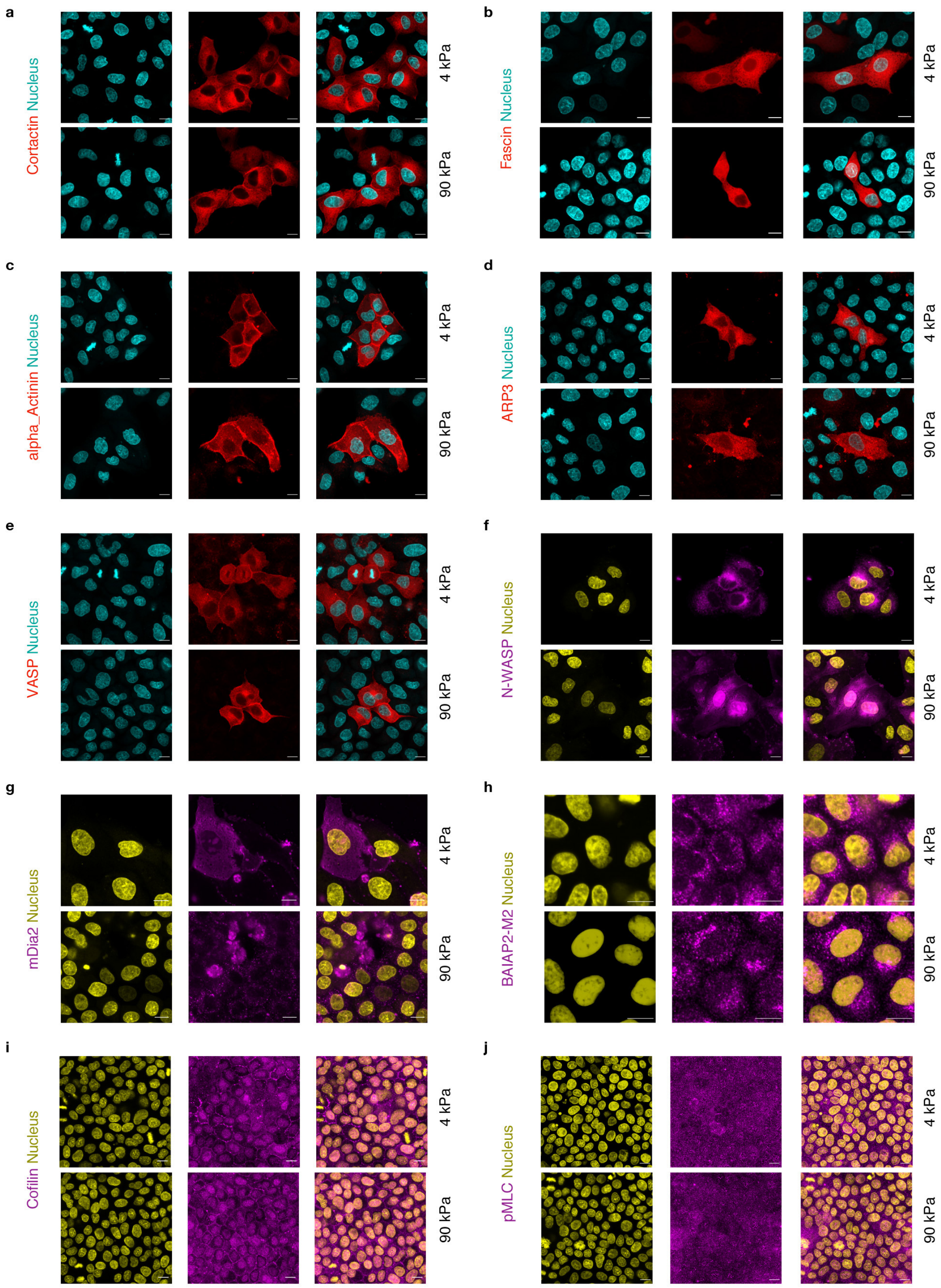

Supplementary Figure 2. ECM-stiffness dependent intracellular localization of actin-binding proteins. a-g. Fluorescence images of MDCK-WT cells transiently transfected with Cortactin_pmCherry (**a**), FusionRed_Fascin (**b**), mApple-alpha-Actinin (**c**), mCherry-ARP3 (**d**), mCherry-VASP (**e**), mEmerald-N-WASP (**f**) and YFP-mDia2 (**g**). **h** Immunofluorescence images of MDCK-WT cells transiently transfected with BAIAP2-M2 and stained with anti-M2. **i,j** Immunofluorescence images of MDCK-WT monolayers stained with anti-cofilin (**i**) or anti-pMLC (**j**). **a-j** Top panels: Soft ECM; Bottom panels: Stiff ECM. Nucleus stained with DAPI. Scale bars, 10μm.

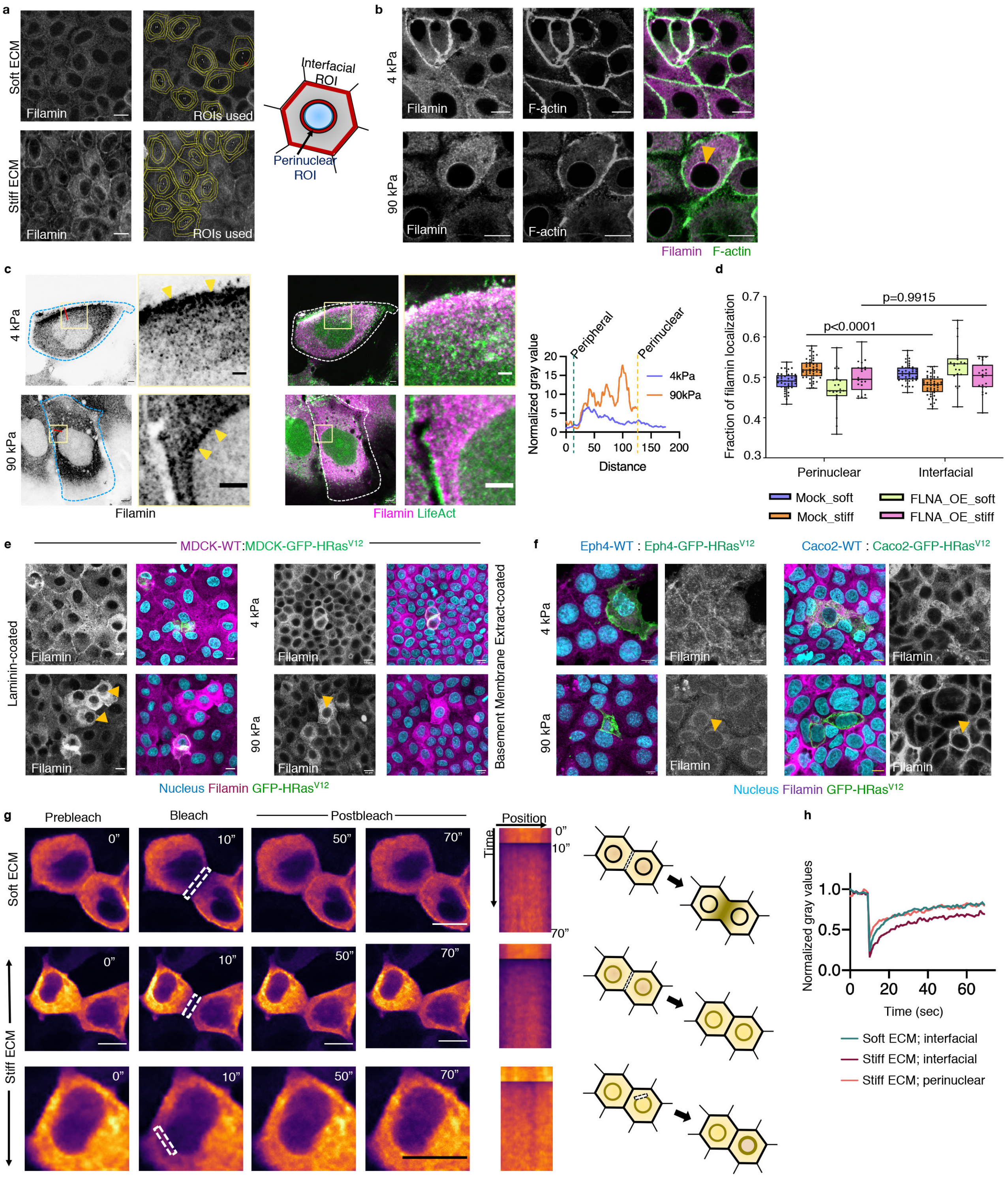

Supplementary Figure 3. ECM stiffness-dependent differential localization and dynamics of filamin. a. Immunofluorescence images of MDCK-WT cells immunostained for filamin cultured on soft (4 kPa, *top* panels) and stiff (90 kPa, *bottom* panels) ECM. Perinuclear and interfacial regions traced manually for quantifying mean fluorescence intensities. Representative grayscale images with traced-out ROIs displayed for cells cultured on soft (*top*) and stiff (*bottom*). Schematic showing the manual ROIs (red) used within each cell for perinuclear and interfacial regions. **b.** Immunofluorescence images of MDCK-WT cells cultured on soft (4 kPa) and stiff (90 kPa) ECM stained with anti-FilaminA and AlexaFluor555-Phalloidin. Yellow arrowhead points to perinuclear co-localization of filamin and F-actin on stiff ECM. **c.** Post expansion images of LifeAct-GFP MDCK cells stained for endogenous Filamin and cultured on either 4 kPa or 90 kPa PAA gels. Filamin localization differences are indicated by yellow arrowheads in the insets. Scale bar, 2 µm; Inset, 1 µm. Fluorescence intensity line scan plots for red line marked in the filamin channel. **d.** Box-and-

whiskers plot depicting the fraction of filamin mean fluorescence intensity at perinuclear and interfacial regions, per cell, on soft (4 kPa) and stiff (90 kPa) ECM for MDCK-WT mosaic and MDCK-mApple-FLNA overexpression mosaic cultures; n = 46, 50, 21, 21 cells over 3 independent experiments. Statistical significance was assessed using unpaired student t-test with Welch's correction (two-tailed). $p(<0.0001) = 6.35542e-09$ For boxplots, centre line denotes median, box displays the interquartile range, whiskers indicate range not including outliers (1.5x interquartile range). Overexpression of mApple-FLNA leads to increased interfacial localization of filamin on stiff ECM. Source data are provided as a Source Data file. **e.** Immunofluorescence images of mosaic cultures of MDCK-WT and transformed cells cultured on soft (4 kPa) and stiff (90 kPa) PAA gels coated with either Laminin (*left*) or ECM-gel (*right*) and immunostained with anti-FilaminA. Yellow arrowheads indicate perinuclear localization of filamin on stiff ECM. **f.** Immunofluorescence images of mosaic cultures of Eph4 or Caco-2 cells transfected with GFP-Ras^{V12} cultured on soft (4 kPa) and stiff (90 kPa) collagen-coated PAA gels and immunostained with anti-FilaminA. Yellow arrowheads indicate perinuclear localization of filamin on stiff ECM. Scale bars: 10 μm. **g.** Photobleaching in mApple- FLNA cells cultured on soft (top panel) or stiff ECM (middle and bottom panels). White dotted- box indicates the photobleached regions: cell-cell interface (*top*, soft ECM), cell-cell interface (*middle*, stiff ECM) and perinuclear region (*bottom*, stiff ECM). Scale bars: 10 μm. Kymographs for the dotted regions shown followed by the schematic depicting the differential and dynamic recovery of filamin. **h.** FRAP curve that depicts the recovery of mApple-FLNA post photobleaching at the ROIs used in (**g**).

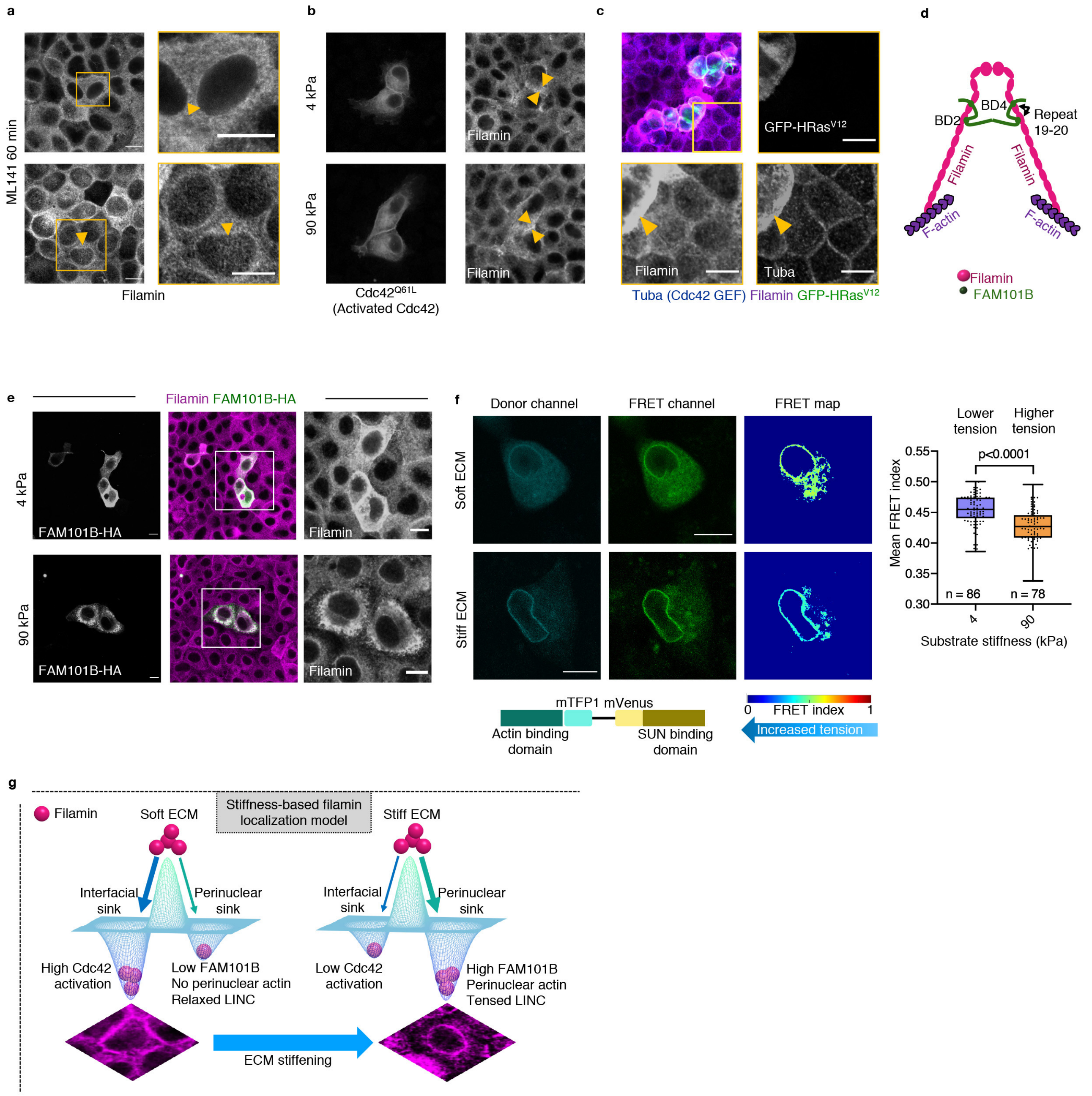

Supplementary Figure 4. FAM101B and perinuclear cytoskeleton regulate perinuclear localization of filamin. a. Immunofluorescence images of MDCK-WT cells cultured on soft ECM treated with Cdc42 inhibitor ML141 (*both panels*) and immunostained for filamin. Yellow arrowheads indicate enrichment of filamin at perinuclear region on soft ECM post treatment with ML141. **b.** MDCK-WT cells transfected with constitutively active Cdc42 (Cdc42^{Q61L}) and immunostained for filamin. Yellow arrowheads indicate increased interfacial enrichment of filamin on stiff ECM. **c.** Immunofluorescence images of MDCK-WT:MDCK-GFP-HRas^{V12} mosaic monolayer co-stained for filamin and Tuba (Cdc42-GEF) on soft ECM. Yellow arrowhead indicates enrichment of Cdc42-GEF at the interface of transformed and WT cells, where filamin accumulates. **d.** Schematic representation for filamin structure, depicting its interactions with FAM101B and F-actin. The filamin-FAM101B-actin localization at perinuclear space is enabled by the complex's interaction with LINC complex, that responds to extracellular force cues. **e.** Immunofluorescence images of MDCK-WT monolayer transfected with FAM101B-HA and stained with anti-HA and anti-Filamin, on soft (4 kPa, *top panels*) and stiff (90kPa, *bottom panels*) substrates. Magnified view of boxed region shown in third panels. FAM101B- HA co-localizes with filamin to perinuclear region on stiff substrate. **f.** Nesprin tension sensor (Nesprin-TS) expressed in MDCK-WT cells cultured on soft (4 kPa, *top panels*) and stiff (90kPa, *bottom panels*) substrates. The schematic underneath shows the construct, where the tension sensor module comprising of mTFP1 and mVenus FRET pair has been inserted between the actin binding domain and SUN binding domain of Nesprin. Increased force increases the distance between the FRET-

pair causing a decrease in the FRET index. (*Right*) Box-and-whisker plot showing significant reduction of FRET index in cells cultured on stiff substrate, indicative of high LINC complex tension. For boxplots, centre line denotes median, box displays the interquartile range, whiskers indicate range not including outliers (1.5x interquartile range). Source data are provided as a Source Data file. **g.** Schematic illustrating the stiffness-based competitive aspect of dynamic filamin localization, being pulled either towards the interfacial sink on soft ECM or towards the perinuclear sink on stiff ECM. Scale bars **(a,b,c,e,f)** = 10µm.

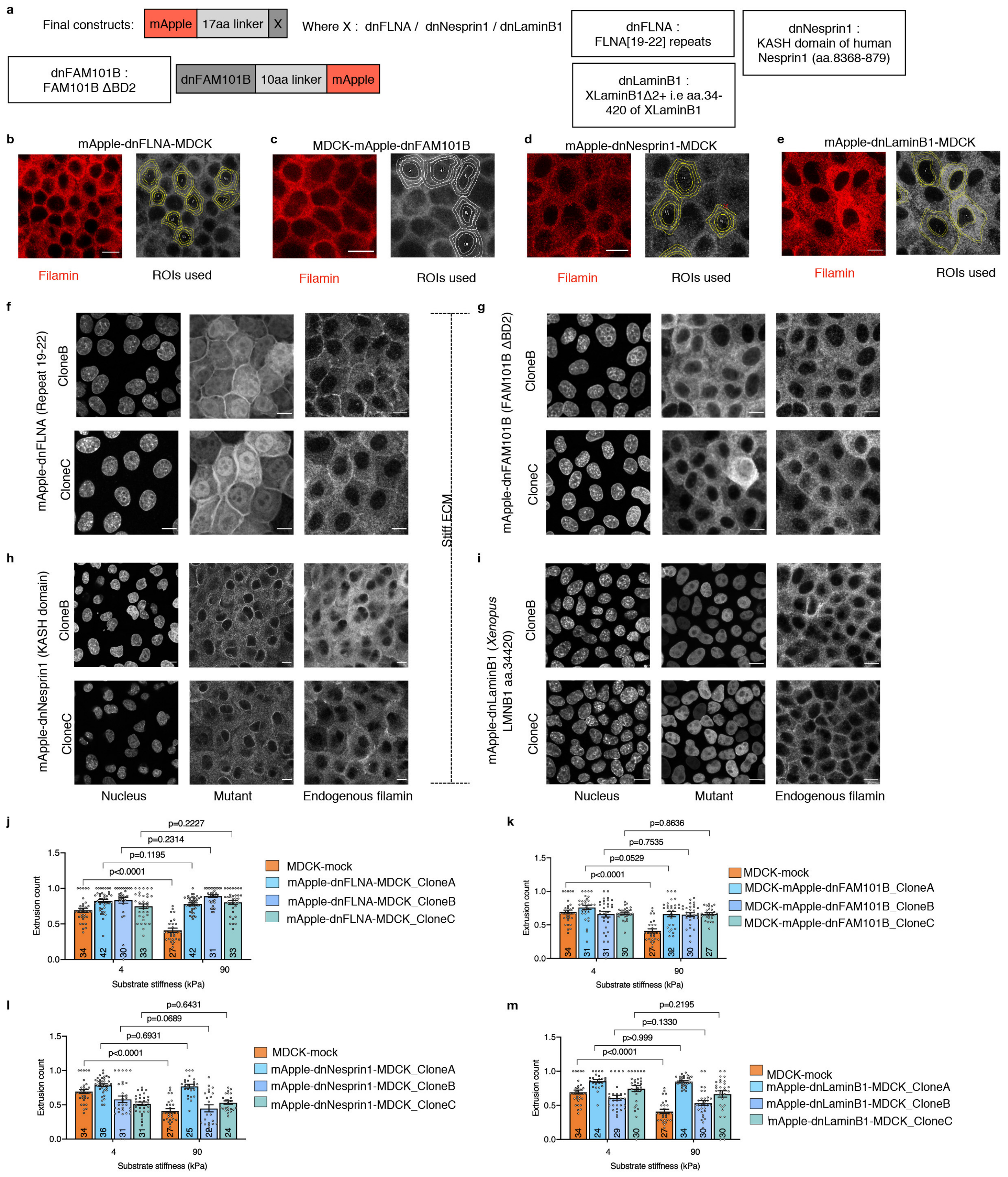

Supplementary Figure 5. EDAC rescue on stiff ECM within MDCK monolayers. a. Schematic representations of dominant-negative mutant constructs used. **b-e.** Immunofluorescence images of mApple-dnFLNA-MDCK cells (**b**), MDCK-mApple- dnFAM101B cells (**c**), mApple-dnNesprin1-MDCK cells (**d**), mApple-dnLaminB1-MDCK (**e**) stained with anti-filamin and corresponding grayscale images with traced-out ROIs displayed for cells cultured on stiff ECM. ROIs used for quantification of the fraction of filamin localization at perinuclear versus interfacial regions. Scale bars, 10μm. **f-i.** Immunofluorescence images of two clones each of mApple-dnFLNA-MDCK (**f**), MDCK-mApple-dnFAM101B (**g**), mApple-dnNesprin1-MDCK (**h**) and mApple-dnLaminB1-MDCK (**i**) stained for filamin. Scale bars = 10 μm. **j-m.** Scatter bar plots depicting the fraction of GFP-HRas^{V12} expressing colonies extruded over soft (4 kPa) and stiff (90 kPa) substrates for 3 clones of each dominant-negative mutant co-cultured with transformed cells; mApple-dnFLNA-MDCK:MDCK-GFP-HRas^{V12} (**j**), MDCK-mApple-dnFAM101B:MDCK-GFP-HRas^{V12} (**k**), mApple-

dnNesprin1-MDCK:MDCK-GFP-HRas^{V12} (**l**) or mApple-dnLaminB1-MDCK:MDCK-GFP-HRas^{V12} (**m**) mosaic populations. Stable expression of dnFLNA, dnFAM101B, dnNesprin1 or dnLaminB1 in surrounding cells rescued extrusion of transformed populations on stiff substrate. Data are mean±s.e.m. collected over 3 independent biological replicates. Statistical significance was assessed using Unpaired t-test with Welch's correction (two-tailed). Significance value (when $p<0.0001$) $p=9.27612e-09$ The number of colonies counted is indicated inside each bar. Source data (**j-m**) are provided as a Source Data file.

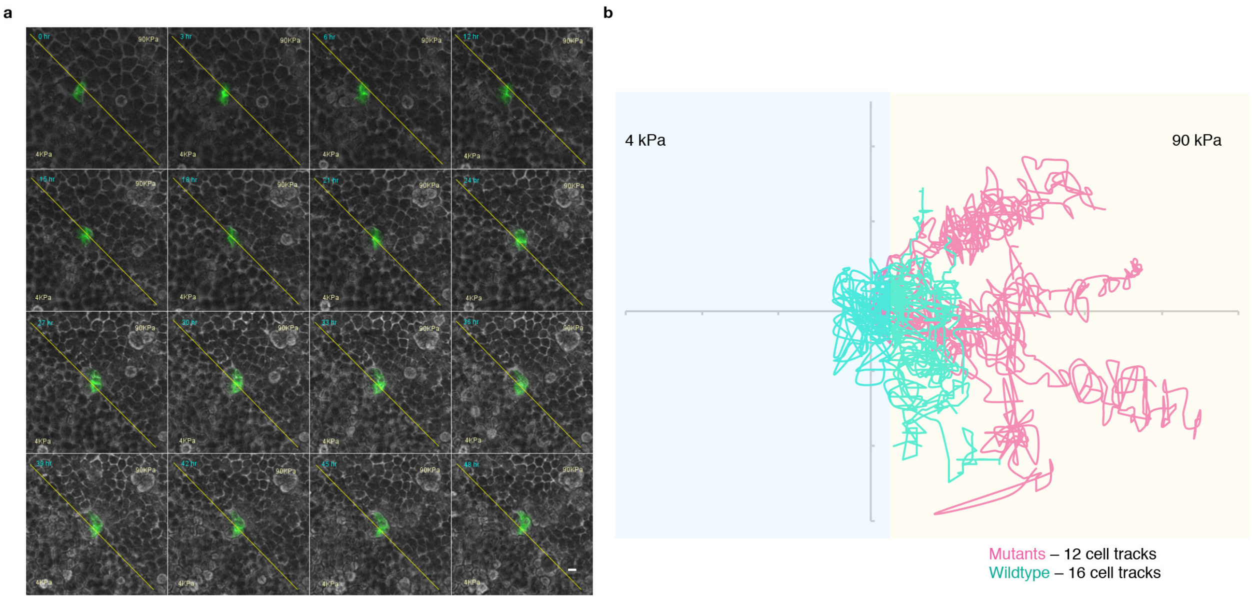

Supplementary Figure 6. Dynamics of transformed-cells on mosaic epithelium. a Representative live imaging time stamps for a competing mosaic monolayer of MDCK-WT and MDCK-GFP-HRas^{V12} seeded on a hybrid 4 kPa and 90 kPa PAA gel. The images depict an HRas^{V12} clone at the interface of this hybrid. Yellow partitioning line marks the exact interface. Scale bar = 10 µm. **b.** Position tracking for 16 MDCK-WT cells and 12 MDCK-HRas^{V12} cells depict non-random migration of HRas^{V12} mutants towards the stiffer substrate as opposed to seemingly random migration of the WT cells. All tracked cells were at the interface of 4 kPa and 90 kPa PAA hybrid gels. The tracks are derived from 4 independent experiments.

Supplementary Table 1. Composition of compliant polyacrylamide gels with different stiffness.

**Stiffness (Young's Modulus)**	**1.2 kPa**	**4 kPa**	**11 kPa**	**23 kPa**	**35 kPa**	**90 kPa**
**milli-Q H_2O** (μl)	820	807	697	695	650	542
**40% acrylamide** (μl)	137	125	250	187	200	300
**2% bis-acrylamide** (μl)	25	50	35	100	132	140
**Fluorescent beads** (μl)	12	12	12	12	12	12
**10% APS** (μl)	5.75	5.75	5.75	5.75	5.75	5.75
**TEMED** (μl)	0.25	0.25	0.25	0.25	0.25	0.25

Supplementary Table 2. Antibody information

Antibodies/Fluorophore	Catalog No.	Manufacturer	Dilution/Working concentration
DAPI (4′,6-diamidino-2-phenylindole)	D1306	Invitrogen	1 μg ml^{-1}
Rabbit anti-DNMBP (Tuba)	ab88534	Abcam	10 μg/ml
Alexa-Fluor-647 Phalloidin	8940	CST	1:40
Alexa-Fluor-555 Phalloidin	8953S	CST	1:40
Mouse anti-FilaminA (monoclonal)	F6682, clone PM6/317	Sigma	1:100
Rabbit anti-HA-tag (monoclonal)	3724S, clone C29F4	CST	1:800
anti-FLAG-M2 (monoclonal)	F1804	Sigma	1:500
Rabbit anti-Cofilin (monoclonal)	5175S, clone D3F9	CST	1:200
Rabbit anti-pMLC2(Ser19)	3671	CST	1:50
Alexa Fluor Plus 594 donkey anti-mouse IgG secondary antibody	A32744	Invitrogen	1:100 when used with anti-FilaminA
AlexaFluor 488, Goat anti-Mouse IgG (H+L) Cross-Adsorbed Secondary Antibody	A-11001	Invitrogen	1:100 when used with anti-FilaminA
Alexa Fluor 488, Goat anti-Rabbit IgG (H+L) Cross-Adsorbed Secondary Antibody	A-11008	Invitrogen	1:200 when used with anti-Cofilin<br>1:50 when used with anti-pMLC2
Anti-rabbit IgG (H+L), F(ab')2 Fragment (Alexa Fluor®555 Conjugate)	4413S	CST	1:100 when used with anti-DNMBP (Tuba)
Anti-rabbit IgG (H+L), F(ab')2 Fragment (Alexa Fluor-647 Conjugate)	4414S	CST	1:800 when used with anti-HA-tag

Supplementary Table 3. Plasmid information

Plasmid	Source	Identifier	Comment
pcDNA4/TO/GFP-RasV12	Yasuyuki Fujita	DOI: 10.1038/ncb1853	Tetracycline-inducible $HRas^{V12}$ expression
Cortactin-pmCherryC1	Addgene	27676	Cytoskeletal protein
FusionRed-Fascin-C-10	Addgene	56117	Cytoskeletal protein
mApple-Alpha-Actinin-19	Addgene	54865	Cytoskeletal protein
mCherry-ARP3-C-12	Addgene	54981	Cytoskeletal protein
mCherry-VASP-5	Addgene	55151	Cytoskeletal protein
mEmerald-N-Wasp-C-18	Addgene	54199	Cytoskeletal protein
YFP-mDia2	Addgene	25420	Cytoskeletal protein
pECE-M2-BAIAP2 wt	Addgene	31656	Cytoskeletal protein
mApple-FilaminA-N-9	Addgene	54901	Filamin overexpression
mApple-N1	Addgene	54567	Destination vector for mutant sequence
mApple-C1	Addgene	54631	Destination vector for mutant sequence
FAM101B-HA	This work	N/A	Human FAM101B and mScarlet cloned into pcDNA3.1+/C-HA
mApple-dnFLNA	This work	DOI: 10.1073/pnas.1104211108	Human FLNA Repeat [19-22]-HA cloned into mApple-C1
mApple-dnFAM101B	This work	DOI: 10.1073/pnas.1104211108	Human FAM101B ΔBD2 cloned into mApple-N1
pcdna nesprin TS	Addgene	68127	Nesprin tensor sensor
pcdna nesprin HL	Addgene	68128	Nesprin headless control
mApple-dnNesprin1	This work	DOI: 10.1242/jcs.02471	aa. 8369-8749 of human Nesprin-1 cloned into mApple-C1
mApple-dnLaminB1	This work	PMID: 11683386	XLaminB1Δ2+ (34-420 aa from X06344) cloned into mApple-C1
