# Supplementary material for: Matrix mechanics regulates epithelial defence against cancer by tuning dynamic localization of filamin
Source: Nat Commun. 2022 Jan 11;13:218. doi: 10.1038/s41467-021-27896-z (PMC8752856; doi:10.1038/s41467-021-27896-z)
Supplement: Supplementary file 3 — Description of Additional Supplementary Files [file 41467_2021_27896_MOESM3_ESM.docx]

**Description of Additional Supplementary Files**

**Title: Supplementary Movie 1. Extrusion of HRas^V12^-expressing cells by normal cells post- induction with doxycycline.**

**Description:** HRas^V12^-expression was induced by doxycycline and tracked up to 23 hours by taking images at 1 hr interval. *Top panels*: DIC images; *bottom panels*: fluorescence images.

**Title: Supplementary Movie 2. Long term dynamics of HRas^V12^-expressing cells in competition with normal cells under hindered extrusion.**

**Description:** Competing mosaic monolayer of MDCK-WT and MDCK-GFP-HRas^V12^ was seeded on 90 kPa PAA gel and observed upto 60 hours post induction with doxycycline. *Left panel*: Phase contrast images; *Right panel*: fluorescence images.

**Title: Supplementary Movie 3. Dynamics of photo-converted mEos2-filamin molecules on soft ECM.**

**Description:** mEos2-filamin cell interfacing with an HRas^V12^ cell was stimulated and tracked for 180 seconds. This video corresponds to the snapshots showed in Fig. 2f, *top panels*, on soft ECM.

**Title: Supplementary Movie 4. Dynamics of photo-converted mEos2-filamin molecules on stiff ECM.**

**Description:** mEos2-filamin cell interfacing with an HRas^V12^ cell was stimulated and tracked for 180 seconds. This video corresponds to the snapshots showed in Fig. 2f, *bottom panels*, on stiff ECM.

**Title: Supplementary Movie 5. Live-imaging of competing heterotypic MDCK monolayer on a hybrid PAA gel.**

**Description:** Live imaging time series and simultaneous tracking of a representative competing HRas^V12^ mutant clone and surrounding WT cells in a mosaic monolayer with MDCK-WT and MDCK-HRas^V12^ seeded on a hybrid 4 kPa and 90 kPa PAA gel observed upto 48 hours post induction with doxycycline.
