# Supplementary material for: Matrix mechanics regulates epithelial defence against cancer by tuning dynamic localization of filamin
Source: Nat Commun. 2022 Jan 11;13:218. doi: 10.1038/s41467-021-27896-z (PMC8752856; doi:10.1038/s41467-021-27896-z)
Supplement: Supplementary file 9 — Reporting Summary [file 41467_2021_27896_MOESM9_ESM.pdf]

nature research

Corresponding author(s): Tamal Das
Last updated by author(s): Dec 6, 2021

Reporting Summary

Nature Research wishes to improve the reproducibility of the work that we publish. This form provides structure for consistency and transparency in reporting. For further information on Nature Research policies, see our Editorial Policies and the Editorial Policy Checklist.

Statistics

For all statistical analyses, confirm that the following items are present in the figure legend, table legend, main text, or Methods section.

n/a	Confirmed	
☐	☒	The exact sample size (n) for each experimental group/condition, given as a discrete number and unit of measurement
☐	☒	A statement on whether measurements were taken from distinct samples or whether the same sample was measured repeatedly
☐	☒	The statistical test(s) used AND whether they are one- or two-sided<br>*Only common tests should be described solely by name; describe more complex techniques in the Methods section.*
☒	☐	A description of all covariates tested
☐	☒	A description of any assumptions or corrections, such as tests of normality and adjustment for multiple comparisons
☐	☒	A full description of the statistical parameters including central tendency (e.g. means) or other basic estimates (e.g. regression coefficient) AND variation (e.g. standard deviation) or associated estimates of uncertainty (e.g. confidence intervals)
☒	☐	For null hypothesis testing, the test statistic (e.g. F, t, r) with confidence intervals, effect sizes, degrees of freedom and P value noted<br>*Give P values as exact values whenever suitable.*
☒	☐	For Bayesian analysis, information on the choice of priors and Markov chain Monte Carlo settings
☒	☐	For hierarchical and complex designs, identification of the appropriate level for tests and full reporting of outcomes
☒	☐	Estimates of effect sizes (e.g. Cohen's d, Pearson's r), indicating how they were calculated

Our web collection on statistics for biologists contains articles on many of the points above.

Software and code

Policy information about availability of computer code

Data collection	Leica Application Suite X (LAX, v3.7.0.20979) and Olympus FV31-SW (v2.3.1.198) software were used to acquire images.
Data analysis	ImageJ1.53e/FIJI was used to contrast and overlay images as described in the Methods section. Statistical analyses were carried out in GraphPad Prism9 (Version 9.2.0) and MATLAB2019b.

For manuscripts utilizing custom algorithms or software that are central to the research but not yet described in published literature, software must be made available to editors and reviewers. We strongly encourage code deposition in a community repository (e.g. GitHub). See the Nature Research guidelines for submitting code & software for further information.

Data

Policy information about availability of data

All manuscripts must include a data availability statement. This statement should provide the following information, where applicable:

- Accession codes, unique identifiers, or web links for publicly available datasets
- A list of figures that have associated raw data
- A description of any restrictions on data availability

The authors declare that all quantifications supporting the findings of this study are available within the paper and its Supplementary Information files, and are provided as a Source data file. Other data, such as raw and processed microscopy images, are available from the corresponding author upon request.

Field-specific reporting

Please select the one below that is the best fit for your research. If you are not sure, read the appropriate sections before making your selection.

- [x] Life sciences
- [ ] Behavioural & social sciences
- [ ] Ecological, evolutionary & environmental sciences

For a reference copy of the document with all sections, see nature.com/documents/nr-reporting-summary-flat.pdf

Life sciences study design

All studies must disclose on these points even when the disclosure is negative.

Sample size	No statistical methods were used to pre-determine sample-size. At least 10 image frames were picked per sample in each biological replicate (for 3 independent biological replicates) for quantification purposes. The only exception was for the experiment involving Raichu-Cdc42 FRET biosensor, where ~5 image frames were picked per biological replicate (for 3 independent replicates). For in vitro experiments the n numbers were determined according to the minimal number of independent biological replicates that significantly identify an effect. Every figure legend has information on exact n for the respective experiment.
Data exclusions	No data was excluded from the analyses.
Replication	The reproducibility of all experiments displayed through representative images was confirmed using at least three biological replicates.
Randomization	Image frames of co-cultured cells used for imaging and quantification were selected randomly and there was no sub-sampling done. Cells were measured randomly within each condition and under every treatment condition.
Blinding	Blinding was considered to be not necessary due to clear effects being observed from experimental data. Thresholds for detecting extruded cells were chosen for each image-frame based on cross-verification from different image planes with objective properties that appeared indistinguishable across conditions.

Reporting for specific materials, systems and methods

We require information from authors about some types of materials, experimental systems and methods used in many studies. Here, indicate whether each material, system or method listed is relevant to your study. If you are not sure if a list item applies to your research, read the appropriate section before selecting a response.

Materials & experimental systems

n/a	Involved in the study	
[ ]	[x]	Antibodies
[ ]	[x]	Eukaryotic cell lines
[x]	[ ]	Palaeontology and archaeology
[x]	[ ]	Animals and other organisms
[x]	[ ]	Human research participants
[x]	[ ]	Clinical data
[x]	[ ]	Dual use research of concern

Methods

n/a	Involved in the study	
[x]	[ ]	ChIP-seq
[x]	[ ]	Flow cytometry
[x]	[ ]	MRI-based neuroimaging

Antibodies

Antibodies used	Dilution and source information of all primary and secondary antibodies are given in Supplementary Table 2.
Validation	All used antibodies are commercially available and have been validated for the application by the respective manufacturer. The validation information is available on the the suppliers homepage.

Eukaryotic cell lines

Policy information about cell lines

Cell line source(s)	MDCK-wild type and MDCK-GFP-HRasV12 were a gift from Yasuyuki Fujita. Eph4-EV and Caco-2 were obtained from ATCC.
Authentication	Original cell lines (MDCK-wild type and MDCK-GFP-HRasV12, Eph4-EV or Caco-2) were authenticated at source. Derived cells (stable cell lines: MDCK-mApple-FLNA; mApple-dnFLNA-MDCK; mApple-dnNesprin1-MDCK; mApple-dnLaminB1-MDCK; MDCK-mApple-dnFAM101B) were not authenticated.
Mycoplasma contamination	All cell lines tested negative for mycoplasma contamination.

Commonly misidentified lines<br>(See ICLAC register)	No commonly misidentified cell line was used
